# Construction and Verification of a Fibroblast-Related Prognostic Signature Model for Colon Cancer

**DOI:** 10.3389/fgene.2022.908957

**Published:** 2022-07-14

**Authors:** Zhe Zhao, Wenqi Li, LiMeng Zhu, Bei Xu, Yudong Jiang, Nan Ma, LiQun Liu, Jie Qiu, Min Zhang

**Affiliations:** ^1^ Zhengzhou KingMed Center for Clinical Laboratory Co. Ltd., Zhengzhou, China; ^2^ Department of Newborn Infants, Children’s Hospital of Nanjing Medical University, Nanjing, China; ^3^ Department of General Surgery, Zhongshan Hospital, Fudan University, Shanghai, China

**Keywords:** single-cell RNA-sequencing(scRNA-seq), colon cancer, cancer-associated fibroblasts (CAFs), immune infiltration, drug sensitivity

## Abstract

Traditionally, cancer-associated fibroblasts (CAFs), an essential component of tumor microenvironment, were exert a crucial part in colon cancer progression. In this study, single-cell RNA-sequencing (scRNA-seq) data from 23 and bulk RNA-seq data from 452 colon cancer patients were extracted from the GEO database and TCGA-COAD and GEO databases, respectively. From single-cell analysis, 825 differentially expressed genes (DEGs) in CAFs were identified between each pair of six newly defined CAFs, named enCAF, adCAF, vaCAF, meCAF, erCAF, and cyCAF. Cell communication analysis with the iTALK package showed communication relationship between CAFs, including cell autocrine, cytokine, and growth factor subtypes, such as receptor-ligand pairs of *TNFSF14-LTBR*, *IL6-F3,* and *IL6-IL6ST*. Herein, we demonstrated the presence and prognostic value of adCAF and erCAF in colon cancer based on CIBERSORTx, combining single-cell marker genes and transcriptomics data. The prognostic significance of the enCAF and erCAF has been indirectly proved by both the correlation analysis with macrophages and CAFs, and the quantitative reverse transcription-polymerase chain reaction (qRT-PCR) experiment based on 20 paired tumor samples. A prognostic model was constructed with 10 DEGs using the LASSO Cox regression method. The model was validated using two testing datasets, indicate a significant survival accuracy (*p* < 0.0025). Correlation analyses between clinical information, such as age, gender, tumor stage and tumor features (tumor purity and immune score), and risk scores revealed our CAF-related model’s robustness and excellent performance. Cell infiltration analysis by xCell revealed that the interaction between CAFs and multiple non-specific immune cells such as macrophages and the dendritic cell was a vital factor affecting immune score and prognosis. Finally, we analyzed how common anti-cancer drugs, including camptothecin, docetaxel and bortezomib, and immunotherapy, such as anti-PD-1 treatment, could be different in low-risk and high-risk patients inferred from our CAF-related model. In conclusion, the study utilized refined colon cancer fibroblast subsets and established the prognostic effects from the interaction with nonspecific immune cell.

## Introduction

Colon cancer is the third most common cancer among women and men and has the second-highest cancer mortality rate worldwide (Siegel et al., 2020a; Siegel et al., 2020b). Even with intensive treatments, the 5-year overall survival (OS) rate of colon cancer is below 60% (Moghimi-Dehkordi and Safaee, 2012). What’s worse, the number of colon cancer patients under 50 years old has been rising sharply in recent years, and the mortality of colon cancer in young men is the highest during 2012–2016 (Wolf et al., 2018; Kasi et al., 2019).

Colon cancer is usually caused by continuous malignant gene mutations and epigenetic changes in the colon and rectum. The tumor microenvironment (TME) plays a vital role and is one of the driving factors in many types of cancer (Kalluri, 2016). The TME is composed of various non-epithelial cells and extracellular matrices. The non-epithelial cells mainly include tumor-infiltrating immune cells, fibroblasts, and vascular endothelial cells. Tumor-infiltrating immune cells, including macrophages, T cells, B cells, NK cells, dendritic cells (DCs), myeloid-derived suppressor cells (MDSCs), and regulatory T cells (Tregs), could affect tumor development and progression through interaction with tumor cells (Quail and Joyce, 2013; Hui and Chen, 2015). Cancer-associated fibroblasts (CAFs), a type of permanently activated fibroblasts, are shown to be important in tumor development and drug resistance (Pietras and Ostman, 2010; Kobayashi et al., 2021). However, the expressions of multiple commonly used fibroblast markers, such as COL3A1 and THY1, vary greatly in different CAF subgroups (Bu et al., 2019). Therefore, in order to develop better colon cancer treatment strategies based on CAFs, new methods are required to better classify CAFs and identify how different CAFs affect tumor development differently. Single-cell sequencing can uncover the cell diversity in tumor tissues in a comprehensive and unbiased manner. In recent years, single-cell Transcriptome sequencing technology has been widely adopted in the study of TME. However, in TME of colon cancer (CC), gene markers for CAFs have not been well elucidated.

**FIGURE 1:**
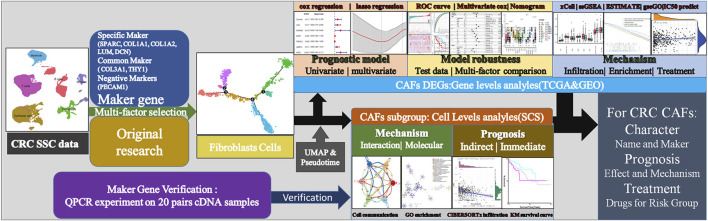
Technical roadmap for this study. We combined experiments and database analysis to reveal the characteristics, prognostic mechanisms and treatment recommendations of CAFs from different perspectives at the gene and cell level.

Over the past decades, technological development in omics and bioinformatics, such as bulk RNA-seq and scRNA-seq, has dramatically advanced the diagnosis and treatment of many types of cancer (Gustafson et al., 2010; Wang et al., 2010). Hae-Ock Lee et al. did scRNA-seq on two colon cancer samples and made their data publicly available (Lee et al., 2020), providing researchers with much information on the characteristics and function of different CAF subgroups. However, due to the high demand for throughput and budget, it is unrealistic to apply large-scale scRNA-seq to a large number of tumor samples. Instead, developing a strategy to explore the valuable information from these existing scRNA-seq data would be more appealing. Furthermore, databases such as TCGA provide us with rich resources, including transcriptomic profiles and clinical information. Combining bulk RNA-seq and scRNA-seq data would be a more time- and cost-efficient approach. Thus, in the current study, we explored the prognosis value of different CAF subtypes in colon cancer patients using TCGA data (i.e., bulk RNA-seq data and OS information) and scRNA-seq data. The CIBERSORTx algorithm was applied to the TCGA bulk RNA-seq data, and the Seurat package was applied to the scRNA-seq data. Finally, we established a CAF-related prognostic signature model that could predict the OS of colon cancer patients. The reliability and accuracy of our weighted model was evaluated comprehensively using the TCGA test dataset and related clinical information. Our model could be well explained by factors such as immune cell infiltration, immune scores, and specific tumorigenic pathways. We further carried out various analyses to make our model accurate for providing suggestion for clinical treatment (Figure 1).

## Results

### Classification of CAFs in Colon Cancer

**FIGURE 2:**
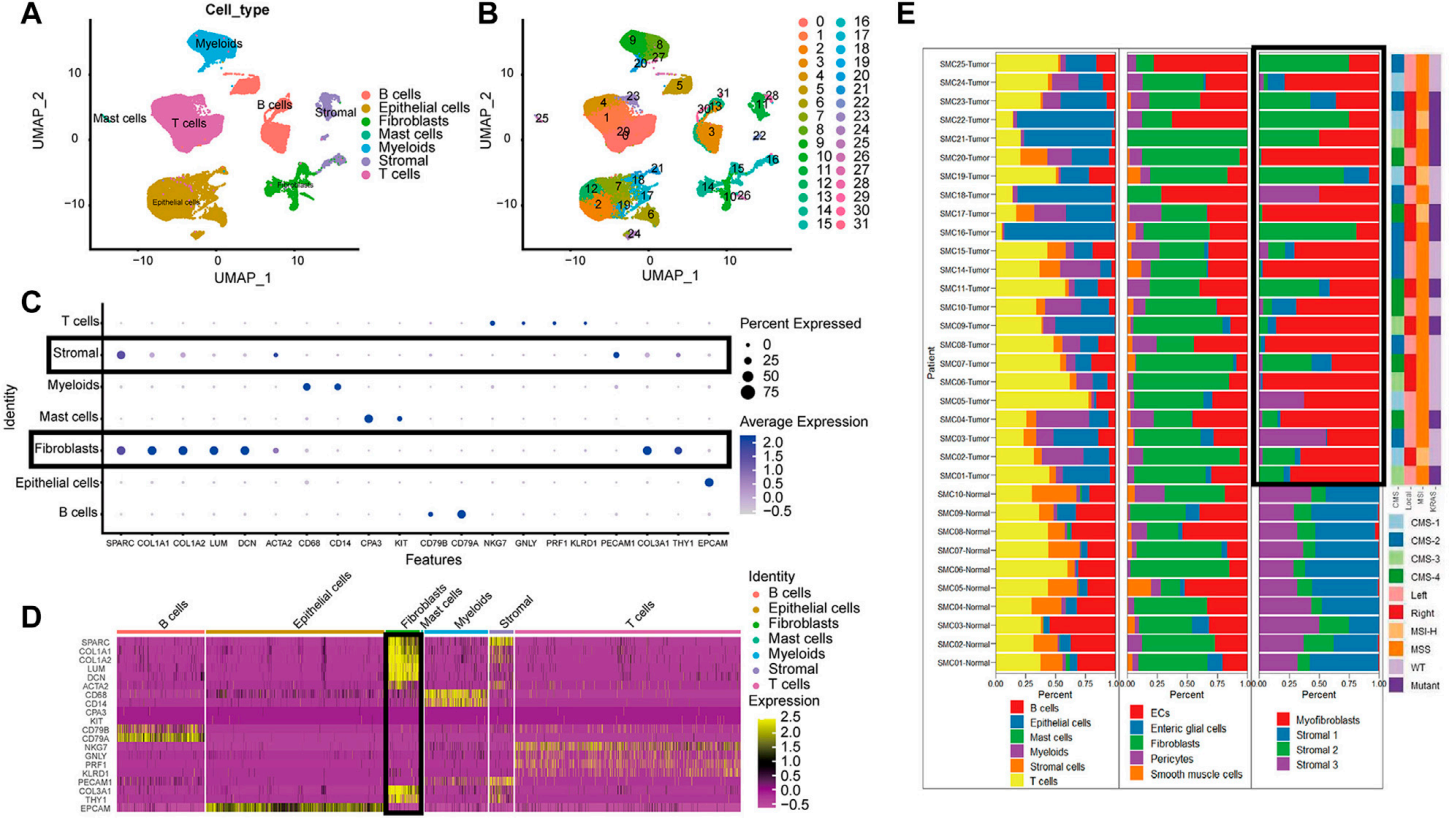
The cell group in CRC based on scRNA-seq. **(A)**. UMAP plot of all cell from the original article **(B)**. UMAP plot of the cluster based on scRNA-seq; **(C)**. Bubble chart of maker gene within all cell type; sizes of dots show gene abundance, while shade shows gene expression level. The main difference of fibroblasts and stromal cells marked with black boxes was the negative selection maker PECAM1. **(D)**. Heatmap of maker gene expression graph for every cell. **(E)**. The proportion structure of all cells, stromal cells and fibroblasts for each patient with clinical information.

The high dimensional information of scRNA-seq enables the identification of CAFs out of a pool of heterogeneous cells, the clustering of CAFs into various subtypes, and the determination of DEGs in different CAF subtypes. In total, scRNA-seq data were extracted for 33 samples, including 23 tumor samples and 10 paracancerous tissue samples from the SMC cohort (GSE132465). After quality control based on the proportion of cell signatures and mitochondrial and ribosomal gene expression, all the cells were classified by the dimensionality reduction algorithms, namely, t-distributed stochastic neighbor embedding (t-SNE) and uniform manifold approximation and projection (UMAP) into seven major and 32 more detailed clusters (Figures 2A,B). According to instructions in the original research, we successfully repeated the stromal cell classification results. We further divided the classified stromal cells into fibroblast and non-fibroblast subgroups. Classification of different cell groups, including the fibroblast subgroup, was validated using a combination of specific gene markers. Common gene markers for CAFs are shown in Figures 2C,D. The CAFs did not express any other cell markers. Specific gene markers, including *SPARC*, *COL1A1*, *COL1A2*, *LUM,* and *DCN*, and common gene markers, including *COL3A1* and *THY1*, were highly expressed in a high proportion of fibroblasts. A low percentage of stromal cells expressed fibroblast gene markers at lower levels, but these stromal cells could be excluded by negative markers of fibroblasts, such as *PECAM1*. The cell types and proportions of different clusters, including stromal cells and fibroblasts, are shown in Figure 2E.

Overall, T cells and epithelial cells (ECs) accounted for a higher proportion, while stromal cells and B cells accounted for a lower proportion, within the tumor samples. Immature ECs and fibroblasts were the dominant cell types in stromal cells. There were significant clustering differences for fibroblasts in tumor and paracancerous tissues (Figure 2E). For instance, there were more activated fibroblasts in the tumors than predominant stromal sub I and III cells in the paracancerous tissues.

**FIGURE 3:**
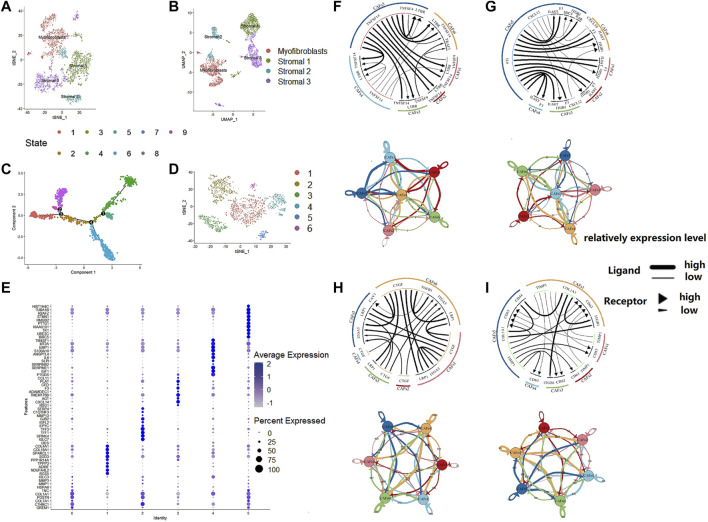
The regroup and cell communication analysis of CAFs in CRC based on scRNA-seq. **(A)**. tSNE plot of the stromal cell from the original article **(B)**. UMAP plot of the stromal cell from the original article **(C)**. The tSNE of pseudotime trajectory analysis **(D)**. tSNE plot showed the regroup of CAFs in CRC based on the pseudotime trajectory analysis. **(E)**. Dot plot for top 10 markers of each CAF subgroup; sizes of dots show gene abundance, while shade shows gene expression level. for the subgroups of CAFs in CRC. The upper parts are the circos plots representing top20 highly expressed ligand-receptor interactions among CAF subgroups; the lower parts are the network plots showing the number of ligand-receptor interactions among CAF subgroups. **(F)**. cytokines/chemokines **(G)**. immune checkpoint genes **(H)**. growth factors **(I)**. others.

We used an unsupervised trajectory analysis to establish a novel classification for the previously classified fibroblast cells. In this approach, we divided all the fibroblast cells in the tumor samples into six subgroups, i.e., CAF1-6, according to the result of an unsupervised trajectory analysis (Figure 3). According to the characteristics of each subtype, we renamed CAF1-6 to enCAF (entoderm-related CAF), adCAF (adhesion-related CAF), vaCAF (vascular-related CAF), meCAF (mesenchyme-related CAF), erCAF (endoplasmic reticulum-related CAF), and cyCAF (cell cycle-related CAF), respectively. The top 10 marker genes for each subgroup are shown in Figure 3E.

### Cell Communicational Signal Analysis and Construction of a Ligand-Receptor Interaction Atlas Among CAFs in Colon Cancer

To analyze the intercellular communication between CAF subgroups, we used iTALK, a cell communication signal analysis tool, to analyze the TCGA colon cancer samples. We explored other factors, including checkpoints, cytokines, and growth factors, which revealed mechanistic insights into the CAF subtype interactions (Figure 3). Many cytokines were identified within the enCAF, acting as the receptor in the intercellular communication. IL6 was most abundant in the erCAF, forming IL6-IL6ST and IL6-F3 receptor-ligand pairs with other CAF subtypes (i.e., erCAF and enCAF). In terms of immune checkpoint genes, *TNFSF14* was highly expressed in erCAF and interacted with other CAF subgroups through the TNFSF14-TNFRSF14 and TNFSF14-LTBR receptor-ligand pairs. Quantitatively, meCAF had more intensive immune checkpoint interactions with other CAF subtypes. Unexpectedly, erCAF only express the receptor, while other CAF subtypes, especially cyCAF and enCAF, could secrete growth factors, such as CTGF, and interact with other CAF subtypes through the CTGF-ITGA5 and CTGF-LRP1 ligand-receptor pairs. Overall, these results showed that the erCAF subtype interacts with the cyCAF subtype *via* the COL1A1-ITGB1 and COL1A1-CD44 signaling pathways; cyCAF subtype also interacts with the other subtypes (i.e., erCAF and enCAF) via the TIMP1-CD63 signaling pathway.

### Functional Enrichment Analysis on Different CAF Subtypes and Association Between CAF Subtypes and Prognosis

**FIGURE 4:**
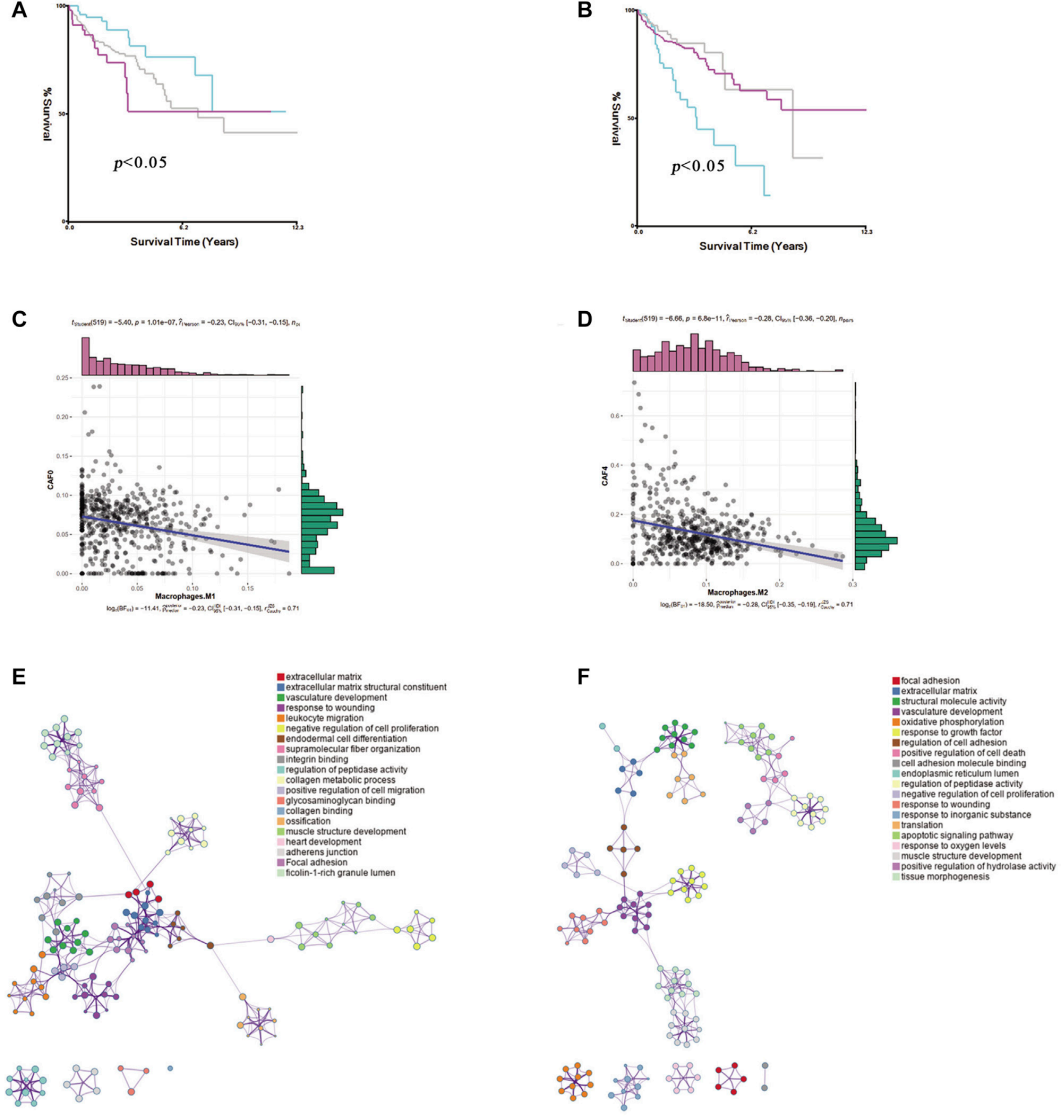
The identification of prognostic CAF subgroup. **(A)**. The Kaplan–Meier plot of the abundance of adCAF, the red line for high abundance, the aquamarine line for low abundance **(B)**. The Kaplan–Meier plot of the abundance of cyCAF, red line for high abundance, aquamarine line for low abundance **(C)**. Figure for the Pearson correlation between enCAF and macrophage M1 **(D)**. The figure for the Pearson correlation between erCAF and macrophage M2 **(E)**. Network diagram for the GO enrichment of CAFs marker genes **(F)**. Network diagram for the KEGG enrichment of CAFs marker genes.

To explore the CAF profiles and understand how different CAF subtypes could affect prognosis in colon cancer patients, we applied the CIBERSORTx algorithm to analyze the abundance of different CAF subtypes and immune cells in colon cancer. We found that the adCAF subtype was a risk factor (Figure 4A) while the cyCAF subtype was a protective factor (Figure 4B) regarding OS of the colon cancer patients. Moreover, we found that the abundance of the enCAF subtype was negatively associated with the abundance of the M1-type macrophages (Figure 4C), while the abundance of the erCAF subtype was negatively related to the abundance of the M2-type macrophages (Figure 4D). To explore the specific function of each CAF subtype, we carried out Gene Ontology (GO) and Kyoto Encyclopedia of Genes and Genomes (KEGG) enrichment analyses, of which the results are shown in Figures 4E,F, and the related -log10 (*p-values*) are shown in Supplementary Figure S1.

Of special note, two key marker genes, *GREM1,* and *IGF1*, were significantly differently expressed in 20 pairs of colon cancer and paracancerous tissues. *GREM1* as enCAF maker gene was highly expressed in colon cancer tissues. On the contrary, *IFG1* as erCAF maker gene was highly expressed in the paracancerous tissues (Figure 9C).

### Construction of a CAF-Related Prognostic Signature Model

**FIGURE 5:**
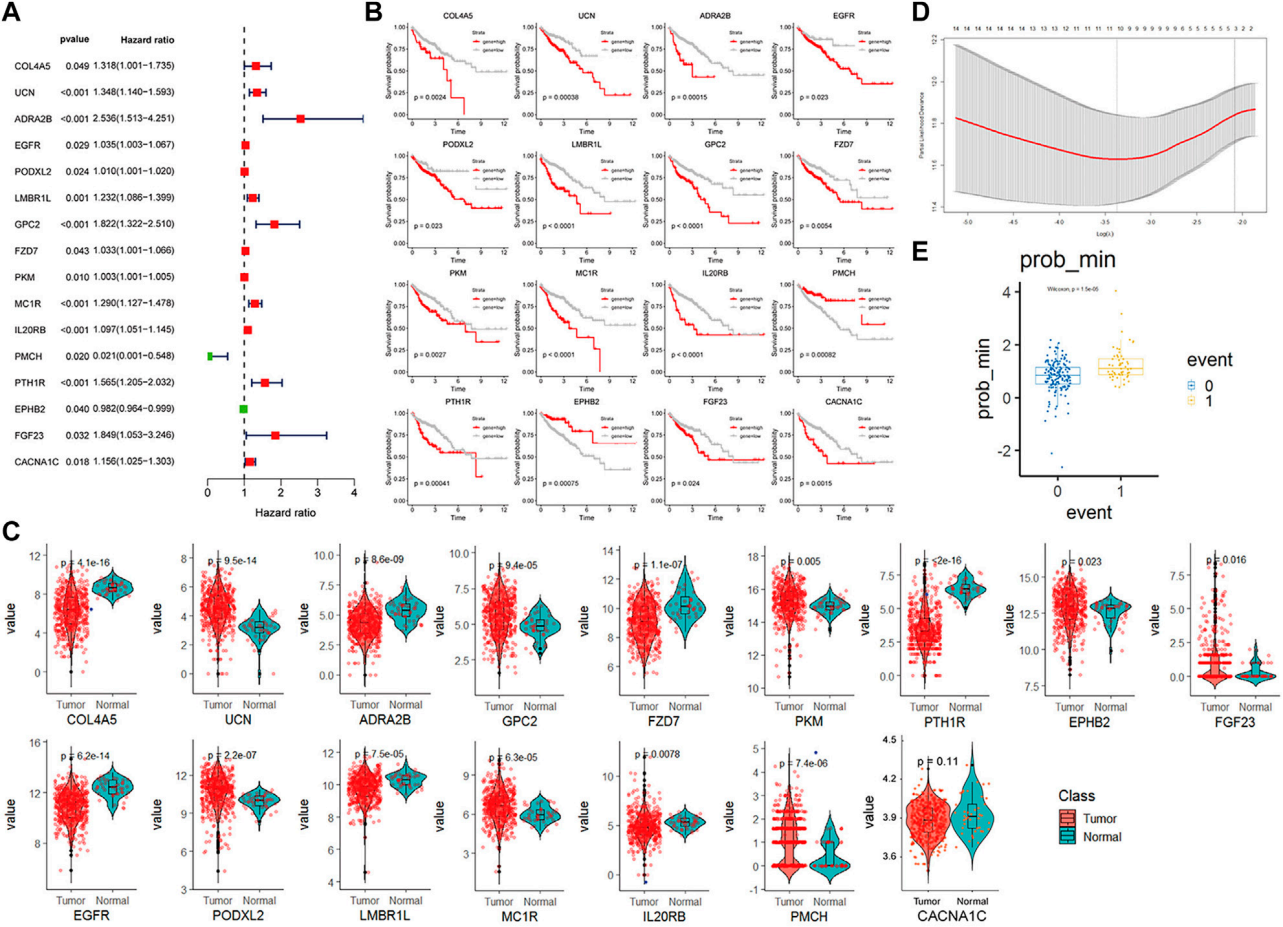
CAF-related gene prognosis model based on TCGA data **(A)**. Forest plot of the 16 genes selected by single factor COX regression **(B)**. The Kaplan–Meier plot of the 16 genes **(C)**. Box plot showing the different expression levels of 16 genes in tumor and normal tissues. **(D)**. Box plot of survival events under lasso optimal regression parameters **(E)**. Number of lasso regression variables genes under the best regression parameters.

We identified 825 highly expressed ligand or receptor genes in different CAF subtypes. To further explore how different CAF subtypes relate to the prognosis of colon cancer patients, we constructed a CAF-related prognostic signature model based on these 825 genes. Fifteen genes that were significantly associated with the prognosis of colon cancer patients were identified by the univariate Cox regression analysis (*p* < 0.05) (Figure 5A). Consistently, expression levels of these 15 genes were significantly different between colon cancer and paracancerous tissues (Figure 5C). The OS was significantly different in the high- and low-expression groups of each of these 16 genes (Figure 5B).

The TCGA colon cancer patients were divided into the training and internal testing datasets by an 8:2 ratio. The least absolute shrinkage and selection operator (LASSO) Cox regression was used to construct the CAF-related signature model. Ten genes were recovered from the LASSO regression analysis under optimal regularization parameters (Figure 5E). Using our model, the OS of patients with colon cancer group by different genes could be well distinguished (Figure 5B). Prognostic genes are weighted by lasso regression, ie the following formula is a simplified weighted model after removing expression correlations between genes. Risk score = (CACNA1C × 0.195) + (COL4A5 × 0.563) + (ADRA2B × 0.734) + (EGFR × 0.082) + (LMBR1L × 0.299) + (FZD7 × 0.119) + (PKM × 0.007) + (IL20RB × 0.384)—(PMCH × 3.74)—(EPHB2 × 0.055). Each gene here represents the transcript expression of the gene (hg38 version), and the coefficient of each gene is the weighted value. The positive and negative values represent tumor-promoting or tumor-suppressing genes, respectively.

**FIGURE 6:**
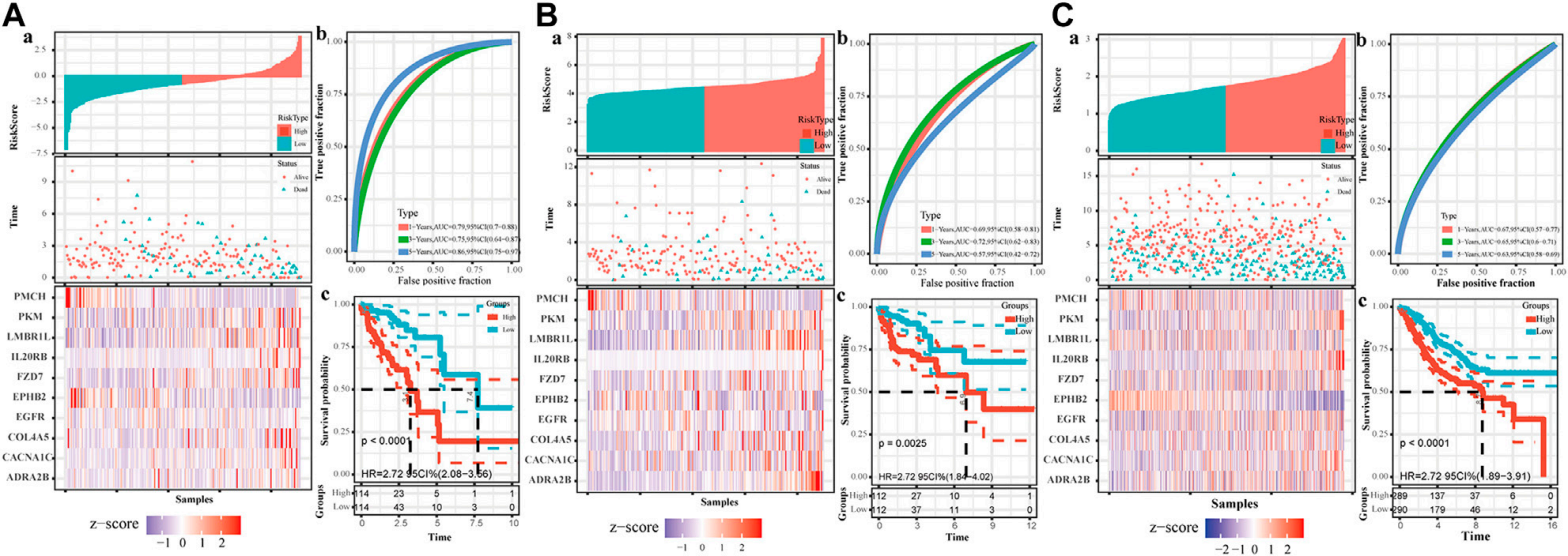
The establishment of CAF-related prognostic signature. The upper left parts are distribution plots for the relationship between risk score and survival status **(A)**; the lower left parts are heatmaps for the expression of 10 genes in the cohort; the upper right parts are ROC curve for the CAF-related signature in the cohort **(B)**; the lower right parts are survival curves between high-risk and low-risk groups **(C)**. **(A)**. Training set **(B)**. Internal testing set **(C)**. External validation set.

Based on the risk model, the patients were divided into high and low scores groups, respectively. Based on this classification, the K-M plot showed a significant difference in high- and low-risk groups in the training dataset (*p* < 0.0001) and the two testing datasets (*p* = 0.0025 and *p* < 0.000, respectively). The area under the curve (AUC) values for OS prediction at 1-, 3- and 5-years of the training dataset were 0.79, 0.75, and 0.86, respectively. Consistently, the AUC values for OS prediction at 1-, 3- and 5-years of the internal and external testing datasets were 0.69, 0.72, 0.57, and 0.67, 0.65, 0.63, respectively, indicating that our signature model is robust and of great prognostic value (Figure 6).

### Accuracy and Robustness of Our Constructed CAF-Related Prognostic Signature Model

**FIGURE 7:**
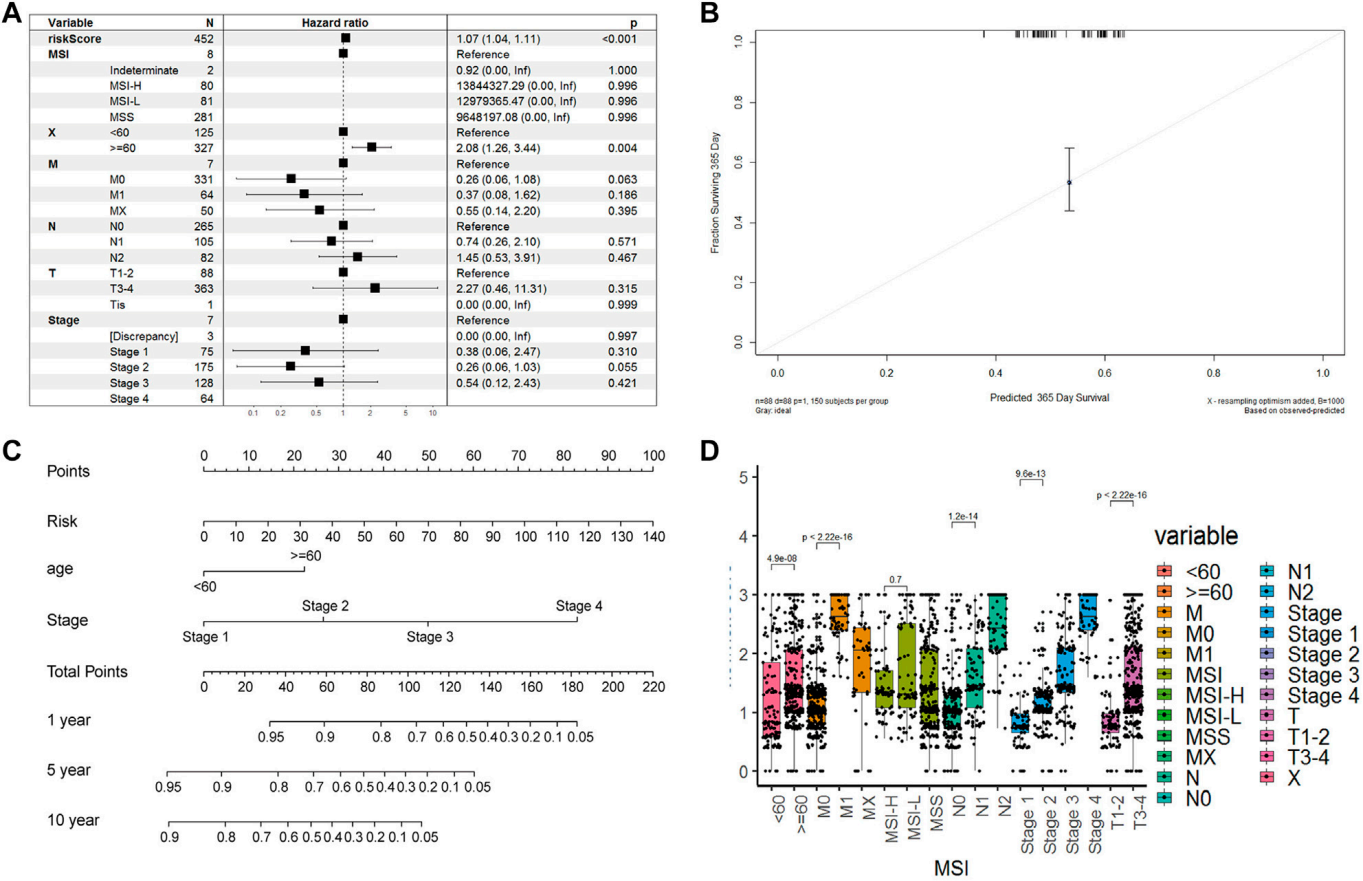
Model robustness analysis based on TCGA clinical information. **(A)**. Forest plot of multivariate COX regression combined with multiple clinical indicators and risk scoring models. Training set **(B)**. Survival nomogram of risk model, age and stade factors **(C)**. The error fitting graph of the risk model under the training set and the test machine **(D)**. Box plot of distribution differences of risk factors in all clinical information groups.

We calculated the risk scores for all patients using our model. The risk scores were different between different subgroups when classifying using different clinical features, including old, N stage, T stage, M stage, and Tumor stage (*p* < 5e-^6^), except for the MSI mutation feature. The distribution of scores was consistent with clinical characteristics. As shown in Figure 7D, patient groups with older age, M1 stage (M staging system), N2 stage (N staging system), stage 4 (tumor staging system), and T3-4 (tumor grade) had higher risk scores. The CAF-related prognostic model performed well, not interfered by multiple clinical factors (Figure 7A). In the multiple Cox regression analysis combining risk scores and clinical factors such as MSI mutation type, patient age, tumor grade, and TNM stage, the prognostic prediction was not affected compared with that from risk scores alone. However, age could contribute significantly to the risk model (*p* = 0.004). Overall, our risk scores correlated better with OS at 1-, 5- and 10-years compared with tumor stage and age (Figure 7B). The calibration curve of the model was very stable, and there were limited variations between the training and the two testing datasets (Figure 7C).

### Possible Molecular Mechanisms Related to Our Prognostic Signature Model

**FIGURE 8:**
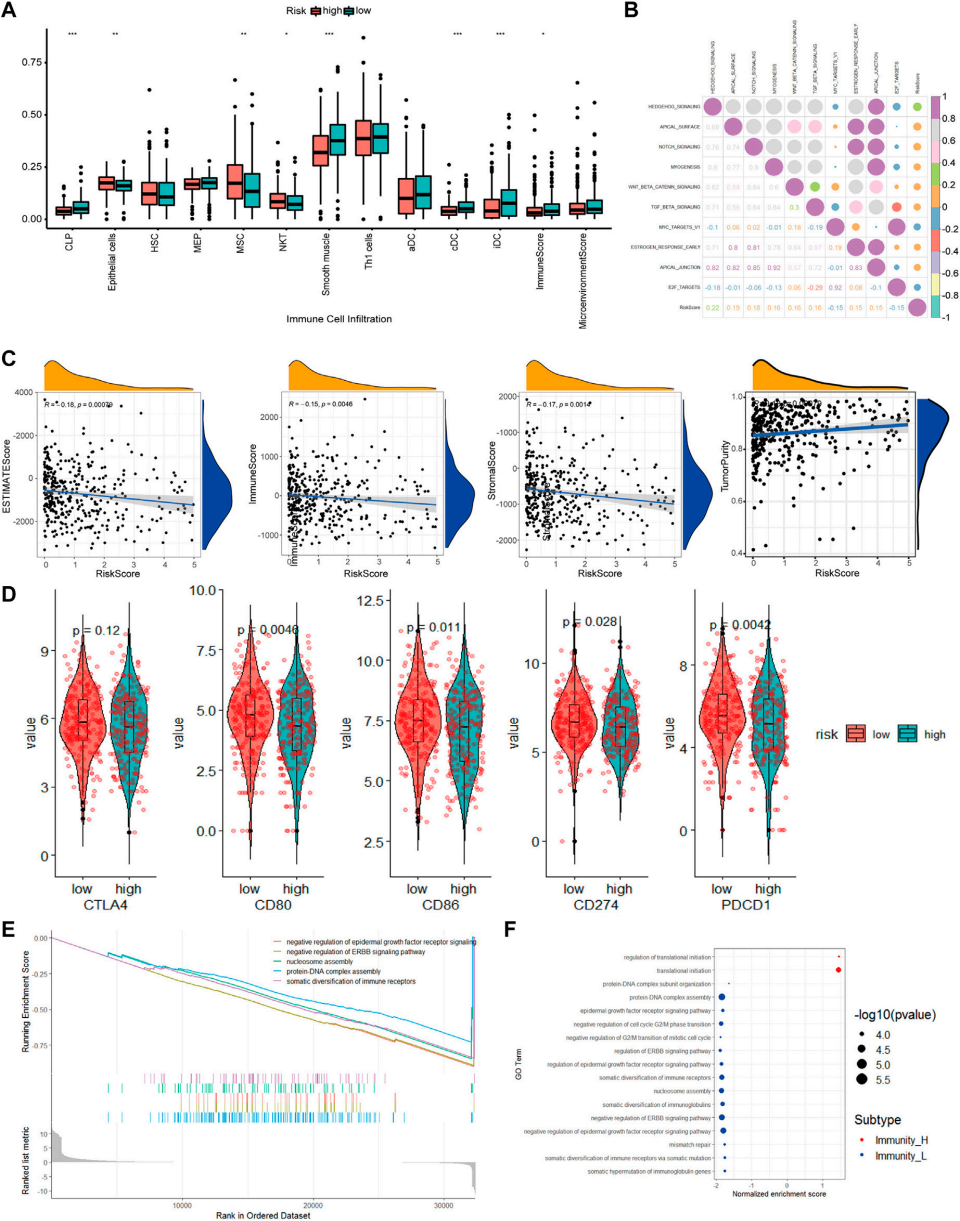
The molecular mechanism of prognostic models based on multiple analyses. **(A)**. Differences in cell infiltration between high and low risk groups based on xCell **(B)**. Correlation heatmap of 50 classic tumor pathways and risk factors **(C)**. Violin chart of the expression levels of immune checkpoint genes in high-risk and low-risk groups **(D)**. Correlation scatter plot of risk factors and multiple infiltration scores **(E)**. The top 5 pathways of gseGO enrichment. **(F)**. Bubble chart of all significant pathways analyzed by gseGO.

To better understand the differences in immune cell infiltration status between different groups classified based on our CAF-related prognostic model, we used xCell to infer the cell infiltration ratio in each sample. Using xCell gene signatures, 11 out of 64 cell types were highly infiltrated with a ratio higher than 5%, including Th1 cells and smooth muscle cells (>25%) (Figure 8A). Of the top seven cell types, epithelial cells and mesenchymal stem cells (MSCs) were identified to be risk factors as these 2 cell types had a high percentage in the high-risk group. On the contrary, common lymphoid progenitors (CLPs), smooth muscle cells, classic dendritic cells (cDCs) and interstitial dendritic cells (iDCs) were identified as protective factors as these cell types had a low percentage in the low-risk group. In addition, immune scores were also significantly different between high- and low-risk groups.

To evaluate how different tumor indicators affect the accuracy of our model, we used ESTIMATE to calculate parameters, including ESTIMATE score, tumor purity, immune score, and stromal score for each of the TCGA-COAD samples and did correlation analyses between these parameters and risk scores. The analyses showed that there were significant correlations between immune scores (R = −0.15, *p* = 0.0046), ESTIMATE score (R = −0.18, *p* = 0.00079), tumor purity (R = 0.18, *p* = 0.00079) and stromal score (R = −0.17, *p* = 0.0014), and risk scores.

Checkpoint-related genes, such as *CD80*, *CD86*, *CD274* and *PDCD1*, were all highly expressed in the high-risk scores (Figure 8D). GO analysis showed many significantly enriched pathways, such as translational initiation, protein-DNA subunit assembly, and G2/M-related cell cycle (Figure 8E). The HEDGEHOG pathway, which is closely related to tumor development, was among the top 10 enriched pathways (Figure 8B). Our model inferred a significant linear correlation among these pathways and risk scores. In the high-risk group, six classical hallmark pathways, including the HEDGEHOG, APICAL, and NOTCH pathways, were highly activated, while two pathways, including the MYCV1 and E2F pathways, were inhibited. As shown in Figure 8B, most of the cancer-promoting pathways showed a strong autocorrelation in Figure 8B.

**FIGURE 9:**
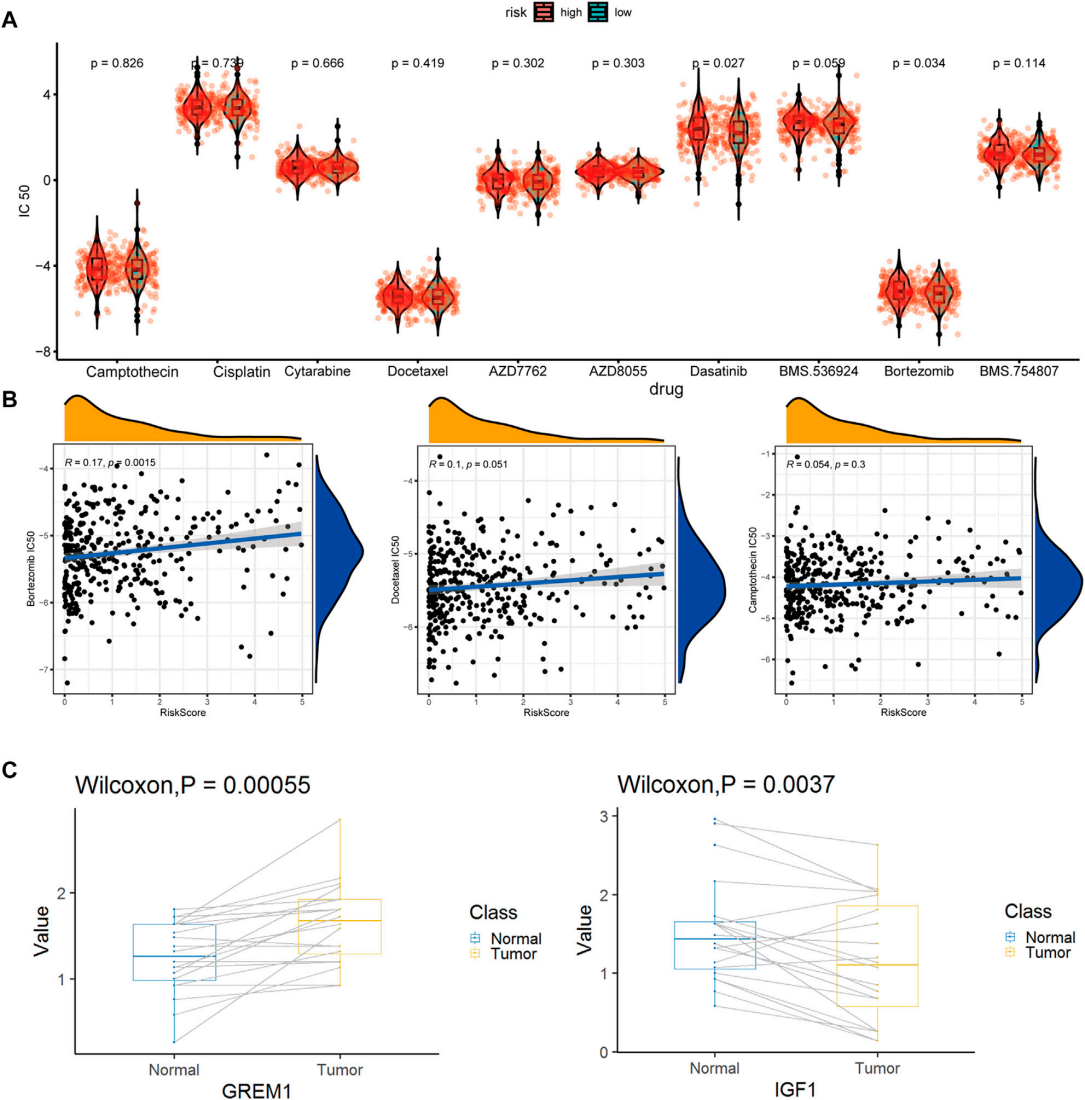
Drug susceptibility prediction and QPCR experimental verification results. **(A)**. Statistical violin chart of IC50 predictions of 10 drugs in high and low risk groups **(B)**. Graph of linear correlation between risk factors and three drugs **(C)**. Box plot of differential expression of key maker genes for enCAF (GREM1) and erCAF (IGF1).

From the CGP database, we identified 10 drugs which was sensitive to colon tumors (Figure 9A). We tested these drugs and identified a total of seven drugs with relatively insignificant IC50 values. Three of the seven drugs, cisplatin, dasatinib, and BMS.536,924, showed poor drug sensitivity, while another 3, camptothecin, docetaxel, and bortezomib, showed strong drug sensitivity. For docetaxel and bortezomib, there was a significant relationship between drug sensitivity and risk scores (Figure 9B), indicating that docetaxel and bortezomib may be more effective in treating low-risk colon cancer patients defined using our model. It is worth noting that the drug sensitivity is more significant in the linear correlation model than in the grouping test. The discrete type of drug sensitivity data in different samples was more significant without an obvious clustering effect.

## Methods

### Data Source

Bulk RNA-seq data and microarray data were downloaded from the TCGA-COAD (Network, 2012) and GSE39582 cohorts (Marisa et al., 2013), respectively. scRNA-seq data (SMC cohort) from 23 colon cancer and 10 paracancerous tissues were downloaded from the GSE132465 cohort (Lee et al., 2020). For bulk RNA-seq data from TCGA, only those with corresponding detailed clinical information were included. As a result, 452 patients from the TCGA-COAD cohort and 579 patients from the GES39582 cohort were included in our study. The TCGA-COAD cohort was used as a training dataset and an internal testing dataset with 8:2 radio. The GSE39582 cohort was used as an external testing dataset. This study followed the guidelines of the TCGA and GEO databases.

### scRNA-Seq Data Preprocessing and Classification of CAF Subtypes

The quality control process was performed using the Seurat R package (version 4.0.1) (Hao et al., 2021). Low-quality cells, which were defined as cells with more than 10% mitochondrion-derived UMI counts, were removed. IntegrateData module in the Seurat was used to eliminate the batch effects among different patients. Here a relatively strict fibroblast system. 1. Preliminary classification of cells group according to the classification method in the original study; 2. Perform final verification according to specific maker and negative selection maker. 3. Perform preliminary verification and statistics according to the common maker of various types of cells (Zhou et al., 2020). The CAF definition in the current study: 1. From tumor samples; 2. Strict Fibroblasts. The CAF subtypes were firstly identified according to the definition in the original article visualized by 2D uniform manifold approximation and projection (UMAP) or t-Distributed Stochastic Neighbor Embedding (tSNE) (Becht et al., 2019).

### Pseudotime Trajectory Analysis

To better classify CAFs, we tried pseudotime trajectory analysis by applying the Monocle 2 R package (Qiu et al., 2017). The “mean expression” parameter was set as > 0.125; the “num_cells_expressed” parameter was set as R10; the *p-value* was set as < 0.01 in the “differentialGeneTest” function. t-SNE plots were used for visualization of the pseudotime trajectories. The 2000 hypervariable genes were selected for analysis, and then the number of principal components (PCs) was set to 20 to obtain cell cluster clusters, and then these clusters were displayed in the form of a “tSNE” diagram. The best category was judged from the number of leaves in the quasi-sequential analysis and the clustering of tSNE.

### Identification of Differently Expressed Genes (DEGs) and Enrichment Analysis

DEGs of the CAF subgroups were identified by the FindMarkers function of Seurat, with cut-offs set as fold change (FC) > 1.5 and adj. *p-value* < 0.01. GO and KEGG enrichment analyses were carried out based on the DEGs, with an adj. *p-value* < 0.05 considered significant.

### Communication Analysis for CAF Subtypes

The identifying and illustrating alterations in the intercellular signaling network (iTALK) R package is a novel tool for intercellular communication analysis based on scRNA-seq (Wang et al., 2019), which could capture highly abundant downregulated or upregulated ligand-receptor gene pairs. We applied iTALK to analyze the ligand-receptor communications among the CAF subgroups and identified a total of 2,648 known ligand-receptor gene pairs. For further analysis, we further divided these gene pairs into four groups, namely, cytokines/chemokines, immune checkpoint genes, growth factors, and the rest.

### Combination of Bulk-Seq and scRNA-Seq Data

CIBERSORTx, which is also known as “digital cytometry”, could infer the proportion of cell types by deconvoluting bulk RNA-seq data (Steen et al., 2020a). We applied CIBERSORTx to estimate the abundance of each CAF subtype in TCGA-COAD patients. We used the software X-tile to set the optimum cut-off values. Patients in each subgroup were divided into CAF-high abundance and CAF-low abundance groups. Univariate Cox regression analysis was performed to analyze the prognostic value of different CAF subtypes in the TCGA-COAD cohort.

### Quantitative Reverse Transcription-Polymerase Chain Reaction

Twenty pairs of fresh colon cancer and paracanceroush tissues from the Fudan University (Shanghai, China) were collected and snap-frozen in liquid nitrogen between October 2020 and September 2021. The samples were then stored at −80°C for later qRT-PCR analysis. In brief, total RNA was extracted using TRIzol reagent (Takara Biotechnology Co., Ltd., Dalian, China). Primers used for qRT-PCR were designed using the Primer5 software. cDNA was prepared using a reverse transcription kit (Takara Biotechnology Co., Ltd.), and qRT-PCR was carried out using the TB Green Premix ExTaq kit and the Applied Biosystems Step One Plus Real-Time PCR system. Ct values were calculated based on housekeeping genes, *ACTB* and *GAPDH*. All the primers were purchased from Takara (Dalian, China) and showed in Supplementary Table S1.

### Construction and Validation of a CAF-Related Prognostic Signature Model for Colon Cancer

The 825 highly expressed DEGs in CAFs were used to construct a prognostic signature model. The univariate Cox regression analysis was performed in the training dataset to identify OS-related genes with *p* < 0.05. Then, LASSO regression analysis was used to optimize the model to avoid overfitting. According to the calculated coefficients from the LASSO analysis, risk score was assigned to each colon cancer patient. Finally, all these colon cancer patients were divided into high- and low-risk groups based on their risk scores, with the median risk score as the cutoff. Kaplan-Meier survival curves and scatter plots were used to visualize OS in the high- and low-risk groups. AUC was used to evaluate the time-dependent predictive accuracy of our model in the training, internal and external testing datasets.

### Independence and Accuracy Test of the Prognostic Signature Model

Multiple cox regression analyses of related clinical factors and risk scores of our prognostic model were based on the “survival” R package. Survival time and survival status, combined with other clinical factors, were used to predict the prognosis by drawing a nomogram established using the “rms” R package, which was then used to illustrate the calibration curve and evaluate the prediction accuracy of the model.

### Immune Infiltration Analysis

The immune score for each sample was calculated using the ESTIMATE package (Steen et al., 2020b). The proportion of different cell types within each tumor sample was calculated using the xCell package with default parameters (Aran et al., 2017). The pathway enrichment score for each sample was estimated using the GSVA package (Hnzelmann et al., 2013). Based on the 50 hallmark pathway feature gene set from MsigDB (H collection) (Liberzon et al., 2015), GO enrichment analysis was carried out by applying the gseGO function of the GSVA package and the clusterProfiler package with “c5. all.v7.1. symbols.gmt” geneset used. For analyses using clusterProfiler, specific parameters were set as follows: ont = “BP”, nPerm = 1,000, minGSSize = 100, MaxGSSize = 1,000, *p*-value cutoff = 0.05. All the significant pathways were shown in the bubble chart, while only the top five significant pathways were shown in the enrichment curve.

### Prediction of Drug Sensitivity

We selected candidate drugs from the CGP database and then applied the pRRophetic package (Paul et al., 2014) to the expression profile of TCGA-COAD samples to predict the drug sensitivity of these candidate drugs. The drug sensitivity of these candidate drugs was further tested in colon cancer cells from the GDSC database (Geeleher et al., 2014). The drug sensitivity is indicated by an IC50 value, which represents the drug concentration when half of the tumor cells die. Low IC50 values indicate better drug sensitivity for colon cancer in our study. It should be noted that the IC50 here is a relative estimate of drug sensitivity. Its value may be less than 0 and does not correspond exactly to the drug concentration.

### Data Visualization and Correlation Analysis

The processing of single-cell data was performed using the Linux platform. Transcriptome data such as those from the TCGA and GEO databases were processed using the Windows10 platform. All the rest analyses were performed using the R 4.0.1 platform. Data cleaning, deformation, and integration were performed using the mgsub, reshape and dplyr packages. Factors such as the cell proportion, risk score, immune infiltration ratio were visualized using the ggpubr and ggplot2 packages (Wickham et al., 2016). The color matching was carried out using the RColorBrewer package (Neuwirth and Neuwirth, 2014). Correlation analysis was implemented using the cor_test and stat_compare_means modules in the R package with default parameters.

## Discussion

### Characterization of CAF Subtypes and Potential Mechanisms in Colon Cancer at the Cellular and Molecular Levels

High-dimensional single-cell RNA-seq data are valuable resources for study at single-cell level. The abundance of fibroblasts is very different between normal and tumor tissues, indicating their importance in tumor development. Traditional bulk RNA-seq is unable to distinguish different CAF subtypes at single-cell level. However, by combining sc-RNA-seq data and bulk RNA-seq data, we were able to identify different CAF subtypes from tumor tissues, such as those from the TCGA database. Using this approach, we successfully identified six CAF subtypes in CRC, which we named enCAF, adCAF vaCAF, meCAF, erCAF, and cyCAF, respectively. We further explored the prognostic significance of these CAF subtypes and discover that two of them, adCAF and cyCAF, were significantly associated with prognosis of colon cancer patients, with the adCAF subtype as a protective factor while the cyCAF subtype as a risk factor. Another two CAFs (enCAF and erCAF) were functioning synergistically and showed an indirect link with prognosis in colon cancer patients. Enrichment analysis revealed that the prognostic significance of enCAF and erCAF related to macrophages. These two subtypes were negatively correlated with the M1 and M2 macrophage infiltration, respectively. M1 macrophages can secrete pro-inflammatory cytokines and chemokines, and present antigens, thus enhancing immune response and surveillance. On the contrary, M2 macrophages can secrete inhibitory cytokines, thus reducing the immune response (Jiawei et al., 2021). Expression levels of these modulating factors were confirmed by qRT-PCR and the key genes in the enCAF and erCAF subtypes were differentially expressed in tumor and paracanceroush tissues. Representative gene *GREM1* was highly expressed in the enCAFs of tumor tissues, which was a risk factor; Representative genes *IFG1* were highly expressed in erCAF of paracanceroush tissues.

To analyze the intercellular communications among the CAF subgroups, we applied the iTALK R package to the scRNA-seq data. Regarding immune checkpoint-related genes, the TNF superfamily member 14 (*TNFSF14*)-lymphotoxin beta receptor (*LTBR*) gene pair was most significantly differentially expressed between the erCAF and other CAF subtypes. TNFSF14 could contribute to vascular and tertiary lymphoid structure formation (Skeate et al., 2020). TNFSF14-LTBR pathway plays a vital role in immune responses in the TME of many types of cancer, but this pathway has not been reported in TME of colon cancer, suggesting it might be an important immunotherapeutic target for CRC treatment. Regarding cytokine-related genes, the *IL6-F3* and *IL6-IL6ST* gene pairs were the most widespread (Figure 3). In several types of cancer, such as breast cancer and hepatocellular carcinoma, CAFs can secret IL6 to promote tumor progression (Dittmer and Dittmer, 2020; Jia et al., 2020). IL6 belongs to a class of polypeptides that can bind to specific, high-affinity cell membrane receptors, regulating multiple cellular functions. The *CTGF*-*ITGA5* gene pair, both encoding growth factors, was differentially expressed between different CAF subgroups. Interestingly, CTGF is a known multifunctional regulator in TME that can activate CAFs, promote angiogenesis and inflammation, thus acting as an oncogene in various types of cancer (Shen et al., 2021). ITGA5 is expressed in CAFs and is responsible for the tumor-promoting effect of CAFs in colon cancer (Lu et al., 2019). Therefore, targeting the CTGF-ITGA5 pathway is promising for colon cancer treatment in patients with a erCAF. Therefore, we have characterized the prognostic significance and potential mechanisms of CAFs in colon cancer at the cellular and molecular levels. Through the detailed description of specific CAF subgroups, the underlying mechanism of CAFs function was indicated, which could be potential therapeutic targets.

In short, we performed deeper bioinformatics analyses, redefined the CAF subtypes, explored the prognostic significance of different CAF subtypes, and carried out experimental validation of key genes in CAFs, to study the role of CAFs in colon cancer development. Other than indices such as tumor size and immune cell infiltration ratio, fibroblast types and ratios may be important prognostic markers for CRC. Understanding the specific roles of different CAF subtypes would be critical for the assessment of prognosis and tumor treatment.

### A Prognostic Signature Model at the Genetic Level

Although we have proven that our CAF-related prognostic signature model is accurate for prognosis assessment and promising for providing treatment recommendations, bulk RNA-seq data were impossible to be applied to this model directly. To further investigate the prognostic value of CAF-related genes, we constructed a CAF-related signature model based on the TCGA-COAD cohort and validated this model using the GSE39582 cohort. With univariate regression analysis, we identified 825 DEGs using scRNA-seq data. Among these DEGs, 16 genes were differentially expressed in colon tumor and paracancerous tissues, which showed excellent prognostic significance in TCGA-COAD patients. Moreover, through lasso regression analysis, we further removed 5 genes that were redundant and thus 10 genes were used as prognostic genes, namely, *CACNA1C*, *COL4A5*, *ADRA2B*, *EGFR*, *LMBR1L*, *FZD7*, *PKM*, *IL20RB*, *PMCH,* and *EPHB2*. A previous study represent that *EGFR* is over expressed in activated CAFs, contributing to colon cancer development (Shin et al., 2019). In addition, some types of CAF from tumors with epithelial-to-mesenchymal transition can escape tyrosine kinase inhibitors (TKIs) mediated EGFR inhibition, suggesting that these types of CAF might relate to EGFR-TKI drug resistance (Mink et al., 2010). In breast cancer, the CAF-derived exosome was able to regulate the expression of *PKM* in cancer cells (Li et al., 2020). However, the associated autocrine signaling in CAFs has not been elucidated. Autocrine signaling-related genes, such as *CACNA1C*, *COL4A5*, *ADRA2B*, *FZD7*, *IL20RB*, *PMCH*, and *EPHB2*, were implicated in some types of cancer (Ikeda et al., 2006; Merlos-Suárez et al., 2011; Kiaii et al., 2013; Phan et al., 2017; Zhang et al., 2018; Cui et al., 2019; Ye et al., 2019).

Next, we validated the established prognostic signature model from the aspects of model effect, test set deviation, and clinical feature comparison. Significant differences were observed regarding the K-M survival curve between high- and low-risk groups in the internal and external testing datasets (*p*≤0.0025). In machine learning on test and training sets, model bias is very limited. In the multiple cox regression analysis, risk factors were significantly correlated with all clinical features (*p* < 5e-^8^) except for the MSI mutation feature. Moreover, the model in the multivariate regression analysis was the substitute for all factors except age, with better prediction range in the nomogram.

In short, comprehensive correlation analyses between multiple prognostic factors and risk scores from our model were performed, and the molecular mechanisms of our model were elucidated. Instead of directly acting on T cells, our prognostic model indicated that CAFs were significantly correlated with CLPs, DCs, and MSCs. CLPs are lymphatic stem cells that can differentiate into T cells, B cells, and NK cells. As a dominant cell type in the intestinal tract, the high ratio of MSCs could explain tumor cells’ low proportion and low activity. DCs are professional antigen-presenting cells (APCs) in the body, where immature DCs can efficiently ingest, process, and present antigens to effectively activate naive T cells, a process important for immune response. MSCs have the tendency to promote tumor development. For example, cytokines secreted by MSCs can inhibit the function of T cells. Probably due to the complex intercellular associations, the risk scores are negatively correlated with the immune scores inferred from multiple scoring algorithms. In addition, as revealed by our model, the prognostic effects relate to 10 classical pathways, including the HEDGEHOG, APICAL, NOTCH, MYCV1, E2F pathways and the translational initiation, protein-DNA subunit assembly, and G2/M-related cell cycle pathways.

In summary, this study established a prognostic model for colon cancer based on CAF-related signature genes, which shows excellent performance compared with models using traditional clinical features. The model is based on the development pathway of cancer and the interaction with various tumor microecological cells to achieve a unified mechanism with key test indicators such as immune score and tumor purity. The model could be a powerful tool for predicting the prognosis of colon cancer patients.

### CAFs on Tumor Development and Treatment

TME is a complex local ecosystem that connects tumors and other parts of the body (Qian et al., 2020). Different from T cells or macrophages that kill tumor cells directly, CAFs play roles in tumor development in an indirect way. Despite the fact that clinical treatment of colon cancer involves many complicated factors, our model could provide potential treatment recommendations based on the transcriptome profile of colon cancer patients. High expressed checkpoint-related genes indicate high activity of immunosuppressive pathways in patients of the high-risk group, who might benefit from treatment of antagonistic antibodies. Importantly, three drugs out of the 10 potential drugs in the GCP database, camptothecin, docetaxel, and bortezomib, may be potential candidates for colon cancer treatment in the low-risk group inferred from our model and may have a better therapeutic effect. In conclusion, starting from the identification of the subgroups of CAFs in colon cancer, by constructing a prediction model for the prognosis of colon cancer patients and prediction of drug sensitivity based on genomics data, the current research was expected to provide new directions and ideas for the CAF-related targeted therapy for colon cancer.

## Data Availability

The datasets presented in this study can be found in online repositories. The names of the repository/repositories and accession number(s) can be found in the article.

## Abbreviations

CAF, cancer-associated fibroblast; DEGs, differentially expressed genes; GEO, gene expression omnibus; GO, gene ontology; KEGG, kyoto encyclopedia of genes and genomes; LASSO, least absolute shrinkage and selection operator; NK cell, natural killer cell; OS, overall survival; scRNA-seq, single-cell RNA-sequencing; TCGA, the Cancer Genome Atlas; ME, tumor microenvironment; t-SNE, t-distributed stochastic neighbor embedding; UMAP, uniform manifold approximation and projection.
