# Supplementary figures and images for: Construction and Verification of a Fibroblast-Related Prognostic Signature Model for Colon Cancer
Source: Front Genet. 2022 Jul 14;13:908957. doi: 10.3389/fgene.2022.908957 (PMC9329609; doi:10.3389/fgene.2022.908957)

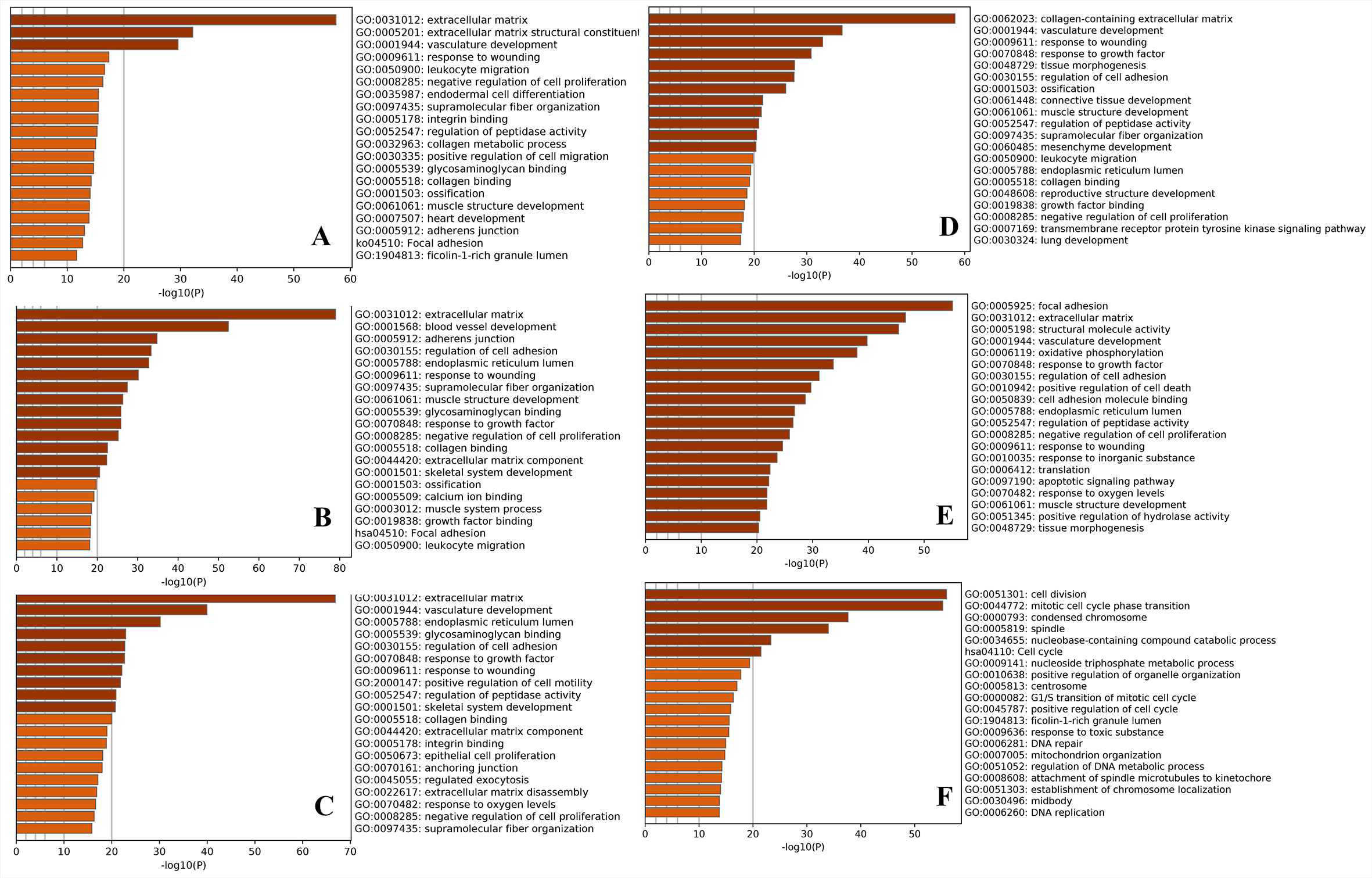

Supplement: Supplementary file 1 [file Image1.TIF]
